# Effect of different origins on genes encoding key enzymes involved in the polysaccharide biosynthetic pathway in *Hedysarum polybotrys* Hand.-Mazz.

**DOI:** 10.1371/journal.pone.0317890

**Published:** 2025-04-22

**Authors:** Zi Xia Wang, Hui Ni Yang, Yan Ping Wang, Yan Jun Jia, Yan Zhang, Li Dong, Fang Di Hu, Guo Lin Chai

**Affiliations:** 1 School of Pharmacy, Lanzhou University, Lanzhou, China; 2 Lanzhou Foci Pharmaceutical Co., Ltd. Research Institute, Lanzhou, China; Tokyo University of Pharmacy and Life Sciences: Tokyo Yakka Daigaku, JAPAN

## Abstract

Radix Hedysari (HP) is a traditional Chinese medicine that has gained widespread attention for its tonic effects. Environmental factors significantly influence the active components in HP, notably Radix Hedysari polysaccharides (HPS), which are the principal active constituents. To date, no studies have reported on the environmental impact on the accumulation and biosynthesis of HPS. This research aims to evaluate the HPS accumulation and its biosynthetic pathways across different environments. We measured the HPS levels in samples from the core geographic area (geo-authentic product region) in Wudu, Gansu Province (WD), and a non-core geographic area (non-geo-authentic product region) in Tanchang, Gansu Province (TC), and conducted transcriptomic analyses. The HPS content in HP from WD (HP-WD, 12.14 ± 0.17 mg/g) was significantly higher than that in HP from TC (HP-TC, 5.48 ± 0.29 mg/g). Our investigation into the biosynthetic pathways of HPS showed that 21 enzymes, encoded by 198 unigenes, are involved. We identified 50 unigenes encoding 15 enzymes as differentially expressed genes (DEGs), indicating that approximately 71.4% of these enzymes are substantially affected by environmental factors. Heat map analysis of these 50 DEGs clearly differentiates HP-WD from HP-TC. Pearson correlation analysis revealed that the regulatory genes for 11 key enzymes have a significant positive correlation with HPS content (*P* <  0.05, *r* >  0.8). Consequently, the HP-WD is more likely to accumulate polysaccharides than HP-TC, potentially due to the activity of the aforementioned 11 key enzymes. This study provides theoretical support for the enhanced HPS content and quality assessment of HP sourced from the geo-authentic product region.

## 1. Introduction

Radix Hedysari (HP), the root of *Hedysarum polybotrys* Hand.-Mazz., is a major medicinal plant in China [1,2], with a medicinal history of more than 1,900 years. In the theory of traditional Chinese Medicine, HP is renowned for its ability to invigorate Yang and tonify “Qi” (vital energy) [3], strengthen exterior and reduce sweat, and nourish body fluid and blood [4]. Modern pharmacological studies have shown that HP exert pharmacological effects on anti-inflammatory [5] and anti-cancer treatments [6], garnering substantial interest across regions including Taiwan, Hong Kong, Macao, and various Southeast Asian countries [7]. Radix Hedysari polysaccharides (HPS) possesses various pharmacological activities and is the main active component in HP [8–10^]^. Notably, HP sourced from the Wudu District is distinguished as genuine medicinal material, and it has been officially designated as a national geographical indication for agricultural products [11]. The synthesis of these polysaccharides, however, is profoundly influenced by environmental conditions. Various abiotic factors like topography [12,13], cold [14,15], salt [16], drought [17] stresses, along with biotic factors including soil microorganisms [18], significantly impact their biosynthesis.

RNA-Seq technology enables the provision of gene expression data in the absence of a reference genome, elucidates gene regulatory mechanisms, and delineates metabolic pathways of compounds. It has been extensively applied in identifying metabolic pathways of compounds and in mining functional genes associated with the biosynthesis of active ingredients [19]. Notable examples RNA-Seq applications in medicinal plants include studies on *Dendrobium moniliforme* (Linnaeus) Swartz [20], *Codonopsis pilosula* (Franch.) Nannf. [21], *Polygonatum cyrtonema* Hua (Liliaceae)) [22], *Bletilla striata* (Thunb.) Reichb.f. [23], *Panax ginseng* C.A. Meyer [24], *Ganoderma lucidum* (Curtis) P. Karst [25]. The biosynthesis pathway of plant polysaccharides can be segmented into three distinct stages: initially, sucrose, synthesized through photosynthesis, is converted into critical monosaccharides like sucrose synthase (SUS), invertase (INV), and hexokinase (HK), which catalyze the formation of uridine diphosphate glucose (UDP-Glu), guanosine diphosphatemannose (GDP-Man) and guanosine diphosphate fucose (GDP-Fuc). Following this, in the cytoplasm, UDP-Glu undergoes further transformation into other NDP monosaccharides like UDP-rhamnose (UDP-Rha), UDP-D-Xylosedisodium (UDP-D-Xyl), and UDP-L-arabinose (UDP-L-Ara). The final stage involves the transportation of these NDP sugars to the Golgi apparatus via nucleotide sugar transporters. Within the Golgi, glycosyltransferases (GTs) attach these monosaccharides to growing polysaccharide chains through a process of dehydration and condensation, culminating in the formation of complete polysaccharide structures. These are then distributed to various tissues within secretory vesicles, where they accumulate [9,14].

This research project is designed to examine the accumulation of HPS and its biosynthetic pathway in different geographical area, which from the core geographical region (geo-authentic product region) in Wudu District of Gansu Province (WD) and non-core geographical region (non-geo-authentic product region) in Tanchang County of Gansu Province (TC). The assessment of HPS levels was conducted using the phenol-sulfuric acid method, a technique noted for its accuracy in quantifying polysaccharide content. Subsequently, the project utilized advanced RNA-Seq sequencing technology to pinpoint and analyze unigenes linked to critical enzymes which involved in the biosynthetic pathway of HPS. This methodology is instrumental in revealing how environmental variables in different geographic locations can affect the production of these important biomolecules.

## 2. Materials and methods

### 2.1 Statement

Experimental materials were collected for the purposes of this study in Wudu District and Tanchang County of Longnan City, Gansu Province. The procurement of these materials in no way affected any endangered or protected species at or around any of the collection locales. The specimens of *Hedysarum polybotrys* Hand.-Mazz. utilised in the investigations were from the established medicinal plant cultivation bases in the WD and TC regions, respectively. It should also be noted that all experimental protocols and methods used during this study strictly adhered to the overall guidelines and law governing plant-based research, insuring that all research carried out within this study was performed responsibly and ethically. This research was carried out at the School of Pharmacy in Lanzhou University.

### 2.2 Plant materials

In this comprehensive study, our fieldwork was concentrated within two strategically selected agricultural sites: Jiaoshan Village, situated within Jiaogong Town of Wudu District (E104°41’01“, N33°33’47”, 2360 m), and Chenjiagou Village, located in Lichuan Town of Tanchang County (E104°15’40”, N34°13’28”, 2524 m). In October 2022, corresponding to the peak harvesting season, fresh roots of wild HPs were systematically gathered. To ensure a robust and replicable dataset, samples from each of the designated areas underwent triple replication, meticulously collected from both core and non-core geographical zones to provide a broad spectrum analysis. The HP were certified by Professor Fang Di Hu (School of Pharmacy, Lanzhou University). Immediately following their collection, these samples were rapidly frozen using dry ice to preserve their biochemical integrity and then transported to our laboratory facilities. There, they were securely stored at an ultra-low temperature of -80°C, maintaining their pristine condition until the RNA extraction phase commenced.

### 2.3 Extraction and content determination of polysaccharides from different geographical area

**Fig 1:**
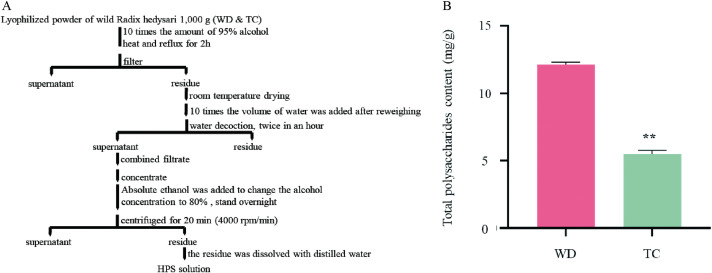
The extraction process and the total polysaccharides content of HPS. (A) The extraction process of HPS. (B) The HPS content of HP –WD and HP-TC (mean ±  SD, n = 3). A t-test was performed for independent treatments, and the “*” is considered significant at ****p**** <  0.01 between the HP–WD and HP-TC. Error bars represent standard deviations.

Our methodology for extracting polysaccharides was adapted from previously established protocols with minor modifications [26]. Initially, the polysaccharides were liberated using boiling water extraction. Subsequently, in our modification, the extract was concentrated to a quarter of its initial volume. We then added anhydrous ethanol until the alcohol concentration reached 80%, and allowed the mixture to stand overnight. The next day, the solution was centrifuged to separate the supernatant, which was discarded, and the precipitate was fully dissolved in distilled water to form a clear HPS solution (Fig. 1A). The content of HPS was performed using the phenol-sulfuric acid method, employing D(+) anhydrous glucose as a calibration standard. This procedure was replicated three times to ensure reproducibility. The correlation between the HPS content and absorbance was depicted in S1 Fig.

### 2.4 Transcriptome analysis

#### 2.4.1 Library preparation and RNA-Seq sequencing.

Total RNA extraction from individual samples was achieved using the RNAprep Pure Polysaccharide Polyphenol Plant Total RNA Isolation Kit, a product of TIANGEN, located in Beijing, China. The RNA’s integrity and yield were evaluated with the aid of an Agilent 2100 Bioanalyzer from Agilent Technologies in Palo Alto, USA. Uniform quantities of superior quality RNA were derived from each sample, which were then subjected to reverse transcription to yield complementary DNA (cDNA). The construction of the cDNA libraries ensued through PCR amplification, complemented by the purification step involving AMPure XP beads. Post-library assembly, an initial quantification was executed using the Qubit 2.0 Fluorometer. To ascertain the insert sizes within these libraries and to ensure precise determination of their effective concentrations, the Agilent 2100 Bioanalyzer was employed. A novel RNA-Seq sequence library was assembled from libraries that had passed quality control, and generated paired-end reads of 150 bp using the Illumina NovaSeq 6000 platform, from Novogene (Beijing, China). The datasets generated during the current study are available in the SRA repository, the BioProject ID is PRJNA1085897.

#### 2.4.2 De novo assembly and annotation of unigenes.

Raw sequence data was filtered in order to guarantee the integrity and reliability of data used for analysis by removing any read containing adapters or having ambiguous base or low quality information. The acceptance of this preliminary cleansing was essential to the purity of the data. Metrics including Q20, Q30, and GC content were computed as an in depth quality cheque for the cleaned sequences. The refined sequences were then de novo assembled using Trinity software, a platform widely proved effective in such complex scenarios. Additionally, the level and completeness of the assembled transcripts was investigated through structural integrity using BUSCO (Benchmarking Universal Single-Copy Orthologs) software, which is highly accurate at quantifying the construction of a given transcriptome.

All assembled genes were identified and functionally annotated by comprehensive homology search against seven public databases-Nr, Nt, Pfam, KOG, SwissProt, KEGG, GO. As a result of this extensive search, the genetic sequences were aligned and annotated to known proteins and functional pathways. To select unigenes from this large dataset, a stringent e-value criterion was determined at a threshold below 10^-5^.

#### 2.4.3 Identification and analysis of differentially expressed genes (DEGs).

The DEseq2 package in R environment (version 3.6.2) was used for the normalisation of the high-quality read sequence. For the concurrent quantification of gene expression values in samples of all origins, the RSEM software (version 1.2.15) was used according to the FPKM values as a standard metric for this quantification [27]. To identify genes with significant differential expression, differential expression analysis was performed with a p value of greater than 0.05 as significant differential expression criterion. These gene expression variations were represented via heat maps generated using the package of the R software (version 3.6.2). In addition, functional annotation and pathway enrichment analyses were carried out on the DEGs using GOseq (version 1.10.0) and KOBAS (version 2.0.12) software for further biological interpretation of the results. They mainly mapped the DEGs onto GO functions and KEGG pathways to find their possible functions by the biological processes and pathways [28,29].

#### 2.4.4 Gene expression was determined by quantitative real-time polymerase chain reaction (qRT-PCR).

qRT-PCR was utilised to substantiate the DEGs in HP from WD (HP-WD) and HP from TC (HP-TC). To ensure the validity and consistency of expression patterns also seen in RT-qPCR gene expression data, both the datasets were thoroughly compared against each other. Total RNAs were then converted to cDNA using a Fast Quant RT SuperMix Kit (item number DP424) by Beijing TIANGEN Biotechnology Co., together with other reagents from Fermentas (MBI) that were supplied by Thermo Scientific Biotechnology Company, USA. This preparatory step was crucial for quantitative analysis in this work. The Quant Studio Design & Analysis fluorescence quantitative PCR system measured quantitative measurements of each gene template through meticulous analyses of Ct values. The 2^-^^△△^^Ct^ method was implemented to calculate the relative gene expression. The relative changes in gene expression between the two groups were then quantified as ΔΔCt values, which are calculated by subtracting the average ΔCt value of the samples for the control from that of the experimental samples. It is highly effective to reveal the subtle genetic variations. Due to both stringent reproducibility, with three biological replicates and three technical replicates for each gene examined, all qRT-PCR assays were conducted. The gene levels were significantly expressed statistically as mean ±  SD, and allow to give a clear and precise measurement of the variability of the expression of these genes in the studied groups. To guarantee the reliability of the qRT-PCR results, this comprehensive approach validated the differential expression patterns established in the first RNA-seq analysis.

## 3. Results

### 3.1 The determination of HPS content in different geographical area

From the experiments, we found that the HPS content in HP-WD (12.14 ± 0.17 mg/g) were significantly higher than that in HP-TC (5.48 ± 0.29 mg/g) (*p* < 0.01). In particular, Fig. 1B shows that the amount of HPS in HP-WD was 2.2 fold higher than that detected in HP-TC. As this pattern shows, HPS accumulates notably more in core geographical areas than in non-core areas.

### 3.2 Transcriptome analysis of the HP-WD and HP-TC samples

Analysis was performed with HP samples derived from HP-WD and HP-TC, which were thoroughly analysed transcriptome using advanced RNA sequencing technologies. Isolation and characterization of genes related to the biosynthesis and accumulation of HPS was the primary objective. This involved several stages: First, raw sequencing data were subject to very stringent filtering to eliminate any artefacts and careful analysis of the sequencing error rates and the distribution of GC content to assure the quality of data used for the subsequent analyses. The details of these preprocessing steps are documented in S1 Table in S1 file. From the processed data, we successfully extracted a total of 135,920,678 clean reads from the combined samples, which demonstrated an average sequencing error rate of 0.03%, a Q30 value of at least 90.52%, an average GC content of 43.26%, and an average mapping rate of 77.8%. Leveraging the power of the Trinity software, we integrated these clean reads to construct a comprehensive HP transcriptome database. From this resource, we were able to identify a total of 81,422 unigenes, each with an average length of 1,104 bp and an N50 value of 2,098 bp. Remarkably, 61.96% (50,453) of these unigenes surpassed 500 bp in length, and 31.83% (25,916) exceeded 1000 bp in length, respectively (S2 Fig.).

**Fig 2:**
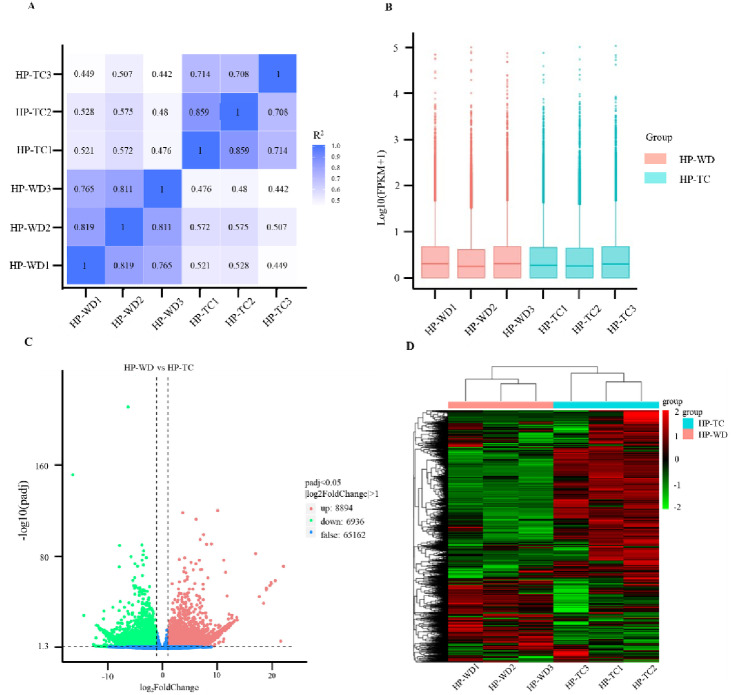
Transcriptome analysis of HP-WD and HP-TC. (A) Pearson correlation analysis of gene expression level. (B) Boxplot of unigenes expressed in the HP-WD and HP-TC with three duplications, respectively. X-axis represents the different ways of growing’s rhizome tissues, and Y- axis shows the log10 (FPKM + 1) values. Significance testing was performed on three samples using the multi-independent sample Krukal-Wallis test. (C) Volcanoplot diagram representation of the number of DEGs identified by transcriptome analysis. (D) Heat map of DEGs. The horizontal coordinate is the clustering result of the sample and the samples, and the vertical coordinate is the clustering result for the differential genes and genes. The color indicates the expression level of the genes in the sample. Darker red represent higher expression level of unigenes, and darker blue represents lower expression level of unigenes.

In this analysis, the correlation coefficients for samples within each group fluctuated between 0.708 and 0.859 (Fig. 2A). The expression levels of genes, were evaluated by computing the FPKM values for HP-WD and HP-TC, revealing that 13,219 unigenes were actively expressed in HP-WD, while 12,788 unigenes were expressed in HP-TC (Fig. 2B). Utilizing DESeq for differential gene expression analysis between the HP-WD and HP-TC, we applied stringent criteria, setting the false discovery rate (FDR) threshold at less than 0.05 and the | log_2_FoldChange | (log_2_FC) at greater than 1. The volcanic plot provided a visual representation of the relationship between FDR and fold change across all targeted genes, facilitating rapid assessment of gene expression variations, illustrated in Fig. 2C. Throughout this study, we successfully identified a total of 15,830 DEGs. Among these, 8,894 genes were found to be up-regulated and 6,936 genes were down-regulated in the HP-WD compared to HP-TC. The heat map cluster analysis distinctly differentiated the genetic expression profiles of HP-WD from those of HP-TC, effectively illustrating the significant contrasts in gene expression between the two sample groups, as shown in Fig. 2D.

### 3.3 Functional annotation and expression of unigenes

Across various genomic databases, a comprehensive annotation of unigenes was performed, encompassing the NR, NT, KEGG, SwissProt, PFAM, Gene Ontology [30], and KOG. The exact numbers of unigenes annotated in each database were 60.40%, 48.86%, 26.83%, 48.88%, 45.91%, 45.3% and 20.92%, respectively, as detailed in S2 Table in S1 File. Moreover, a subset of 10,660 unigenes, representing 13.09 percent, were concurrently annotated across the NR, NT, KEGG, SwissProt and KOG databases, as shown in S3 Fig S1 File. Further analysis through the Gene Ontology database revealed that 36,887 unigenes were specifically annotated, highlighting the most prevalent categories. Within the realm of biological processes, ‘cellular process’ and ‘metabolic process’ emerged as the dominant categories. For molecular functions, ‘binding’ and ‘catalytic activity’ were identified as the most populated categories, demonstrating a significant focus on these functional attributes (S4 Fig in File).

**Fig 3:**
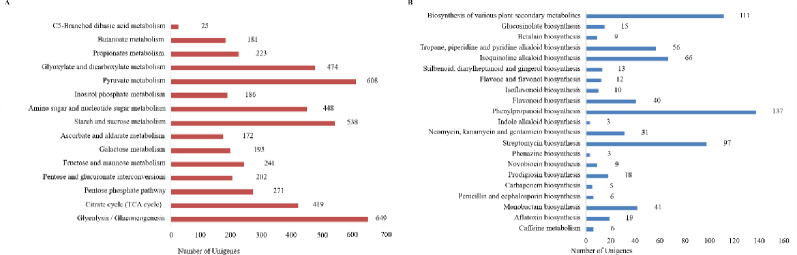
KEGG annotations on HP. (A) Pathway classification of carbohydrate metabolism. (B) Pathway classifications for the biosynthesis of other secondary metabolites.

To thoroughly explore biological mechanisms involved in of HPS biosynthesis, 64,594 unigenes were annotated and linked to 377 distinct pathways within the KEGG database. The most significantly enriched 20 pathways are depicted in S5 Fig in S1 File. Analysis of the DEGs between HP-WD and HP-TC within the KEGG pathways highlighted notable discrepancies, particularly in pathways associated with starch and sucrose metabolism and glycolysis/gluconeogenesis. The subcategory ‘carbohydrate metabolism’ encompassed 15 pathways, with ‘glycolysis/gluconeogenesis’ harboring the highest number of unigenes (649). Furthermore, 1,876 unigenes were implicated in various aspects of polysaccharide biosynthesis, touching on critical metabolic routes such as amino and nucleotide sugar metabolism, fructose and mannose metabolism, glycolysis/gluconeogenesis, and pentose and glucuronate interconversion, as showcased in Fig. 3A. An additional 21 pathways were identified that contribute to the biosynthesis of other secondary metabolites, including pathways like caffeine metabolism (ko00232) and anthocyanin biosynthesis (ko00942), with the phenylpropionic acid biosynthesis pathway containing the largest contingent of unigenes, as illustrated in Fig. 3B. This extensive mapping provides a comprehensive view of the metabolic networks influencing HPS production and other related metabolic activities.

### 3.4 Differential gene expression analysis of the HPS biosynthetic pathway in different geographical area

**Fig 4:**
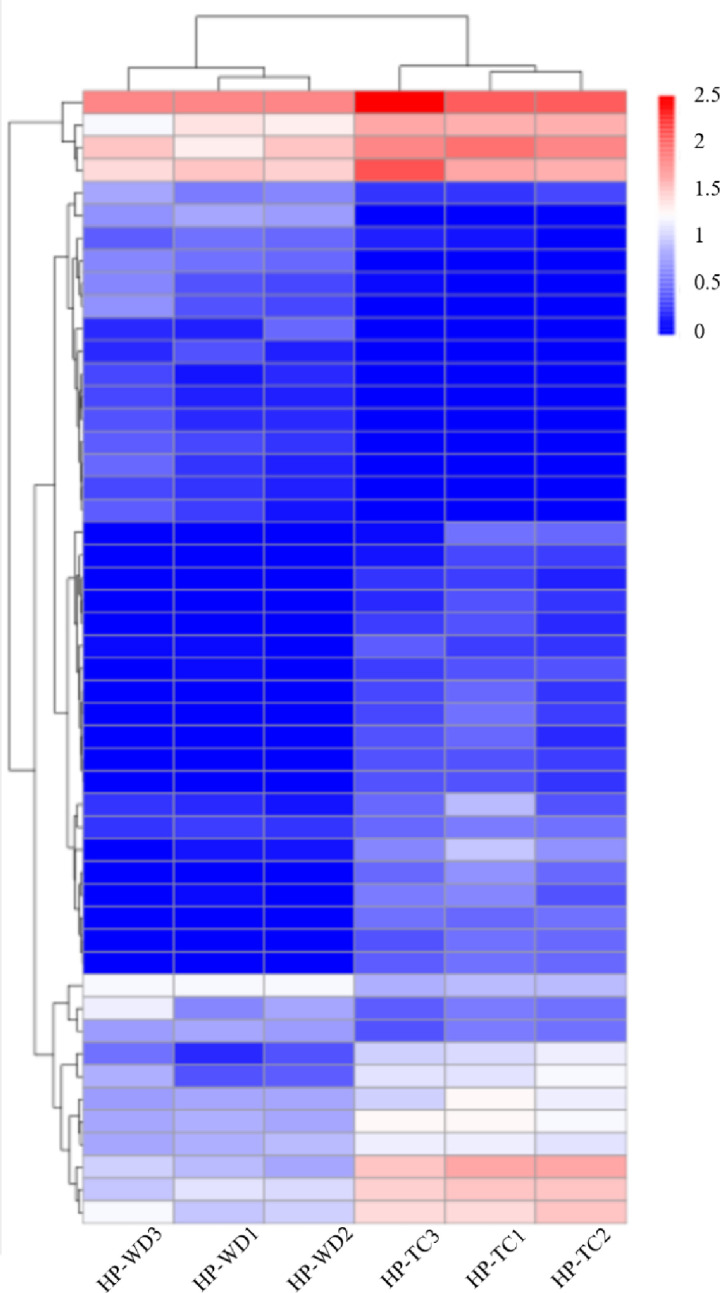
Clustering heat maps of 50 DEGs in HPS biosynthesis pathways from different geographical regions. The horizontal coordinate is the sample and the clustering result of the sample, and the vertical coordinate is the clustering result of differential genes and genes. Darker red represent higher expression level of unigenes, and darker blue represents lower expression level of unigenes.

In our study, we utilized the KEGG database to annotate 1,547 unigenes associated with several key metabolic pathways: amino acid and nucleotide sugar metabolism (ko00520), fructose and mannose metabolism (ko00051), starch and sucrose metabolism (ko00500), and glycolysis/gluconeogenesis (ko00010). Special attention was given to identifying unigenes encoding the critical enzymes within these metabolic routes. Setting a threshold of FPKM greater than 0.3 for our screening criteria, we isolated 198 unigenes that code for essential enzymes such as UDP-rhamnose synthase (RHM), GDP-mannosepyrophosphorylase (GMPP), UDP-glucuronate 4-epimerase (GAE), and others, detailed in Table 1. From these enzyme-encoding unigenes, 50 unigenes of 15 enzymes were further identified as DEGs, indicating that about approximately 71.4% of the enzymes critical for HPS biosynthesis were significantly impacted by environmental variables, as evidenced in S3 Table S1 File. The distinctive expression patterns of these DEGs were effectively visualized through a heat map clustering analysis, which clearly differentiated the HP-WD and HP-TC samples, as shown in Fig. 4. This analysis underscores the significant influence of environmental conditions on the expression of enzymes involved in key biosynthetic pathways, highlighting their potential regulatory roles in the metabolic processes of HPS.

**Table 1:** Number of unigenes encoding key enzymes involved in polysaccharide biosynthesis in HP.

Enzyme name	Abbreviation	EC	NO.	DEG	UP	DOWN
sucrose synthase	SUS	2.4.1.13	8	3	1	2
sucrose-phosphate synthase	SPS	2.4.1.14	5	2	2	0
Hexokinase	HK	2.7.1.1	31	6	2	4
β-fructofuranosidase	INV	3.2.1.26	7	4	2	2
UDP-sugar pyrophosphorylase	UGPase	2.7.7.64	9	4	1	3
Fucokinase	FRK	2.7.1.52	1	0	–	–
glucose-6-phosphate isomerase	GPI	5.3.1.9	17	3	1	2
mannose-6-phosphate isomerase	MPI	5.3.1.8	16	3	1	2
GDPmannose 4,6-dehydratase	GMD	4.2.1.47	1	0	–	–
UDP - glucose 4 - epimerase	UGE	5.1.3.2	12	4	0	4
UDP glucose 6-dehydrogenase	UGDH	1.1.1.22	10	4	3	1
UDP-apiose/xylose synthase	AXS	--	2	0	–	–
UDP-arabinopyranose mutase	UAM	5.4.99.30	4	0	–	–
UDP-glucuronate 4-epimerase	GAE	5.1.3.6	5	2	0	2
GDP-L-fucose synthase	TSTA3	1.1.1.271	1	0	–	–
UDP-glucose 4,6-dehydratase	RHM	4.2.1.76	3	2	0	2
UTP-glucose-1-phosphate uridylyltransferase	UGP2	2.7.7.9	23	15	1	3
Phosphomannomutase	PMM	5.4.2.8	4	2	1	1
mannose-1-phosphate guanylyltransferase	GMPP	2.7.7.13	12	2	0	2
Phosphoglucomutase	PGM	5.4.2.2	24	5	3	2
UDP-arabinose 4-epimerase	UXE	5.1.3.5	3	0	–	–

**Fig 5:**
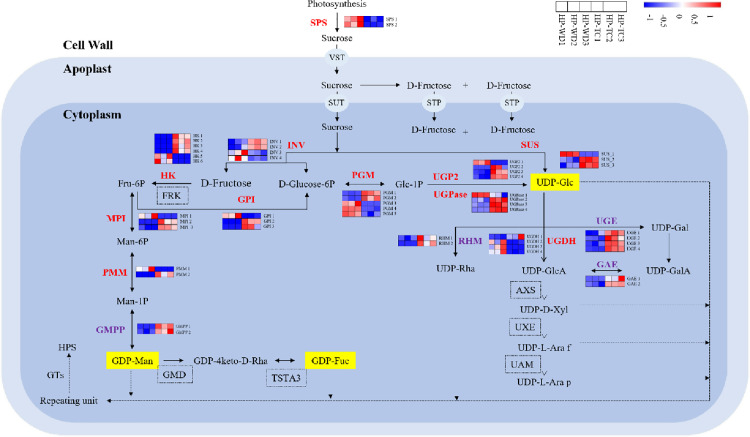
Proposed pathway for the polysaccharide biosynthesis in HP. Activated monosaccharide units, marked in black with yellow background; key enzymes, marked in red or purple. The expression of enzymes in red bold fonts was significantly positively correlated with the content of polysaccharides, while the expression of enzymes in purple bold fonts was significantly negatively correlated with the content of polysaccharides. Various blocks represent the logarithms of FPKM values for different samples (HP-WD1, HP-WD2, HP-WD3, HP-TC1, HP-TC2 and HP-TC3 from left to right). The real line arrows represent the identified enzymatic reactions, and the dashed line arrows represent multiple enzymatic reactions by multiple steps.

In investigating the gene expression patterns of key enzymes involved in the HPS biosynthesis from samples sourced from HP-WD and HP-TC, it was noted that the distinct geographic origins of the plants contributed to unique gene expression profiles. However, the influence of geographic location on the expression levels of genes that regulate these critical enzymes proved to be inconsistent. This variation allowed for the utilization of the FPKM method to accurately pinpoint genes responsible for encoding the enzymes essential for HPS biosynthesis. Employing the transcriptional profiles of these identified enzymes, we meticulously mapped out the potential biosynthetic pathways involved in the formation of HPS. These pathways were comprehensively illustrated in Fig. 5, providing a clear visual representation of the biosynthetic routes potentially responsible for HPS production.

### 3.5 Relationship between HPS content and regulatory genes involved in key enzymes in the HPS biosynthesis pathway

The study analyzed the correlation between the content of HPS and the genes regulating key enzymes in the biosynthesis of HPS, as depicted in S6 Fig in S1 File. & S4 Table S1 File. The analysis revealed that as many as 16 genes demonstrated a significant positive correlation with the polysaccharide content (*p* < 0.05, r >  0.8). Notably, these genes are responsible for encoding a range of critical enzymes involved in HPS biosynthesis, including SPS, INV, HK, GPI, PGM, SUS, UGP2, UGPase, UGDH, MPI, and PMM.

The analysis of HPS biosynthesis across different geographical origins of HP demonstrates that the process is segmented into three distinct phases, which is consistent with biosynthesis patterns observed in other medicinal plants [31]. However, variations in biosynthetic pathways of HPS among different populations are evident. The regulatory genes of 6 enzymes (FRK, GMD, AXS, UAM, TSTA3, UXE) in the HPS biosynthesis pathway exhibited stability, whereas others displayed regional variations, with the expression levels of these genes being regionally correlated. Specifically, in the initial stage of the HPS biosynthesis pathway, the regulatory genes of 10 enzymes, including SPS, INV, SUS, HK, GPI, PGM, PMM, UGP2, MPI and UGPase, were found to have a strong positive correlation with HPS content. During the second step of HPS biosynthesis pathway, key genes that regulate the enzyme UGDH also showed a significant positive correlation with the HPS content. Notably, across the biosynthetic pathway, only the expressions of all regulatory differential expressions of genes for SPS and UGDH consistently exhibited a positive correlation with the content of HPS. This indicates that the first biosynthetic pathway primarily focuses on the UDP-Glc, whereas the second phase is directly catalyzes by UDP-Glc to form various uronic sugars such as UDP-GalA, UDP-D-Xyl, UDP-L-Ara *f,* and UDP-L-Ara *p*. In the final stage, GTs play a crucial role by transferring glycogroups from these activated donor molecules to various receptors, completing the synthesis of diverse polysaccharides.

### 3.6 Validation and expression analysis of genes encoding key enzymes

**Fig 6:**
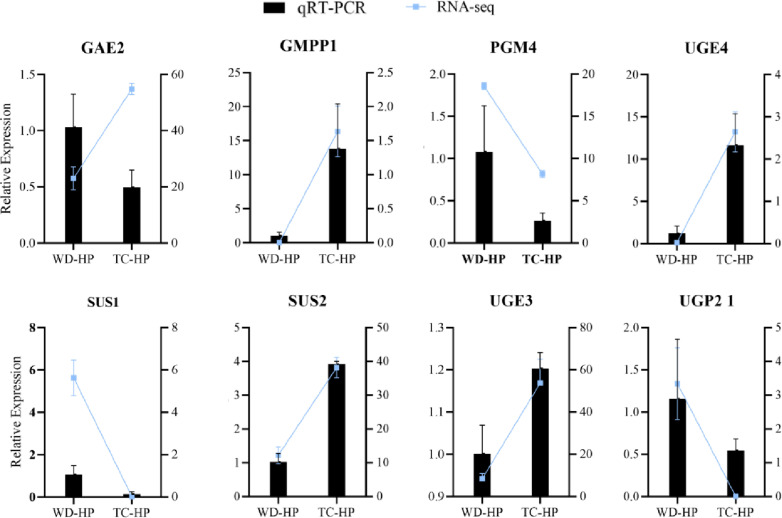
Expression analysis of candidate genes of HP. The expression levels of 8 genes in WD-HP and TC-HP for qTR-PCR and RNA-seq experiment (ment ±  SD, n = 3).

To substantiate the gene expression profiles derived from RNA-Seq sequencing, qRT-PCR was conducted. This analytical technique was applied to assess the expression levels of 8 DEGs, which are responsible for encoding 6 enzymes including GAE, GMPP, PGM, SUS, UGE, and UGP2. These findings are visually represented in Fig. 6. The qRT-PCR results corroborated the RNA-Seq data, affirming that the trends in gene expression observed among these 8 DEGs were consistent across both experimental techniques.

## 4. Discussion

### 4.1 Effect of different geographical area on HPS accumulation

HPS are celebrated for their multifaceted health benefits, including anti-cancer properties [32], gastroprotective effects [33], antioxidant capabilities [34], and other therapeutic effects [6,35]. In this study, to examine how geographical variations affect the accumulation of HPS, we analyzed the HPS content in samples collected from different regions. It was found that the HPS content were substantially higher in HP-WD compared to HP-TC. Literature suggests that HP-WD is associated with enhanced HP-WD has higher antioxidant, immune-boosting, and anti-tumor properties [36]. These observations indicate that the geo-authentic region, known for producing HP-WD, contains significantly higher levels of HPS compared to other regions. This suggests that the superior therapeutic qualities of HP from this core geographic area may be directly linked to higher polysaccharides accumulation.

### 4.2 Effect of origin on HPS biosynthetic

The structural composition and biological activity of polysaccharides are intricately linked [37]. Currently, specific pathways for polysaccharides biosynthesis in medicinal plants from the same family as HP remain undocumented. To deepen our understanding of the biosynthetic pathways of HPS, a comparative analysis was conducted between the known pathways in other documented medicinal plants and those of HPS. During the initial phase of HPS biosynthesis, the conversion of sucrose branches into two distinct pathways: one leads to the production of the key intermediate UDP-Glc, catalyzed by the SUS, and the other leads to D-Glc-6P or D-Fru, INV. Comparative studies reveal that *Hedysarum polybotrys* Hand.-Mazz., *Bletilla striata* (Orchidaceae) [23] and *Polygonatum sibiricum* Red [38] showed stronger INV catalytic action, while *Codonopsis pilosula* (Franch.) Nannf. [21] predominantly utilizes SUS for synthesizing UDP-Glc, whereas Dendrobium officinale demonstrates significant activity of both INV and SUS activity [39].

In the biosynthesis pathway of HPS, the second stage involves synthesizing active monosaccharides. These monosaccharides typically are converted through the UDP-Glc process, resulting in the formation of various sugars including UDP-Gal, UDP-D-Xyl, UDP-L-Ara and UDP-Rha. While pathways for polysaccharide biosynthesis are common across all medicinal plants, the specific pathways vary greatly owing to differences in species and other botanical characteristics. Consequently, these variations account for the differing activities observed in traditional Chinese polysaccharides.

During the third phase of HPS biosynthesis, NDP monosaccharides in their activated forms are incorporated into the growing polysaccharide chain through the action of GT enzymes. This process includes steps of dehydration and condensation to form the final polysaccharide structure. Future research will focus on a detailed examination of the GTs that participate in this pathway, aiming to enhance the understanding and optimization of HPS biosynthesis.

In this analysis, genes related to SPS, PGM, and UGDH are found to be more highly expressed in HP-WD compared to HP-TC, correlating with the notably higher HPS content in HP-TC. Particularly, SPS plays a crucial role in regulating carbon through various physiological and developmental processes such as the starch synthesis and carbohydrate accumulation [40]. The expression level of SPS not only show a significant positive correlation with the HPS content in HP-WD but are also consistent with observations in other species such as tomato [29] and sugarcane. Serving as a key rate-limiting enzyme in the photosynthetic production of sucrose, SPS is directly involved in sucrose metabolism. The activity of SPS is significantly associated with the plant’s resistance to abiotic stresses [40] and influences plant growth and development [41,42]. It is hypothesized that the elevated expression of SPS in HP-WD might relate to the high HPS content, which needs to be verified by further experimental verification.

PGM catalyzes the interconversion between D-Glucose-6P and D-Glucose-1P, playing a critical role in the metabolism of sucrose crucial for the synthesis of cell walls [42]. This enzyme thus bridges the metabolism of polysaccharides with the central carbon metabolism [43]. There exist two forms of PGM: one found in plastids (pPGM) and the other in the cytoplasm (cPGM) [44]. Within chloroplasts, pPGM is responsible for converting Glu-6P to Glu-1P. In contrast, cPGM in the cytoplasm manages the conversion of these glucose phosphates, utilizing Glu-6P primarily for respiration, whereas Glu-1P contributes to sucrose metabolism and cell wall formation. At present, two cPGM isoforms have been reported in Arabidopsis [44], tobacco [45] and maize, while only one cPGM isoform has been reported in potato and spinach [46]. Interestingly, two isoforms of cPGM (PGM2 and PGM3) were found in HP cytoplasm. PGM2 is involved in the conversion of Glu-6P to Glu-1P, while PGM3 is involved in the interconversion of GluNAc-1P. The functions of PGM2 and PGM3 in Arabidopsis are redundant, but the functions of the two cPGM isoforms in HP have not been reported. The levels of intercellular polysaccharides and exopolysaccharides were significantly higher in *Ganoderma lingzhi* overexpressing PGM than in wild-type *Ganoderma lingzhi* [47].

The primary components of higher plant cell walls include polysaccharides such as cellulose, hemicellulose, and pectin [42], with a significant proportion deriving either directly or indirectly derived from UDP-GlcA [48]. UGDH plays a pivotal role in the synthesis of UDP-GlcA, which is essential for constructing cell walls [49,50]. For instance, the heterologous expression of PeUGDH4 in Arabidopsis notably boosted the content of hemicellulose and soluble sugars [51]. The expression of UGDH surpass those observed in both HP-WD and HP-TC, with ongoing studies aimed at determining if this differential expression correlates with variations in cell wall polysaccharide contents. Collectively, this underscores SPS, PGM, and UGDH as crucial enzymes in the biosynthesis pathway of HPS. Consequently, future investigations should concentrate on confirming the hypothesized roles of these genes utilizing advanced molecular biology and related techniques, including proteomics.

Carbohydrate production in plants is influenced by a range of biotic and abiotic stress factors. To support their developmental and growth requirements, plants modulate their polysaccharide levels [24]. The elevated levels of polysaccharides content in HP -WD could be attributed to enhanced photosynthetic activity [52]. Additionally, optimal stress conditions may play a role. Such stresses can trigger starch breakdown, which in turn boosts the content of maltose and glucose, thereby facilitating the buildup of soluble sugars [52,53].

## 5. Conclusion

In this study, significant attention was directed towards the content of HPS, a crucial pharmacological compound found in HP, comparing samples from both core and non-core geographical regions. Analytical results revealed that HPS levels were considerably elevated in samples from the core region. This discrepancy led to transcriptomic analyses, marking the first instance where the biosynthetic pathway of HPS was elucidated, identifying 21 essential enzymes. The study revealed that geographic factors distinctly influence the expression of genes regulating 15 of these enzymes. Furthermore, a strong positive correlation was observed between the gene expression of these 11 enzymes and the levels of HPS. Verification through quantitative real-time PCR on 8 genes affirmed the RNA-Seq data, reinforcing the notion that geographic authenticity enhances the quality and HPS concentration in HP. This investigation underpins the theoretical basis for the superior HPS concentration in geo-authentic regions of HP production.

## Supporting information

S1 File S1 Fig. Standard curve of glucose at 490 nm. S2 Fig. Length Distribution of unigenes and transcripts. S3 Fig. Venn diagram of annotated unigenes from the different databases. S4 Fig. Gene Function Classification (GO) of HP. S5 Fig. KEGG pathway enrichment of the differential genes in HP-WD and HP-TC. S6 Fig. Pearson correlation analysis between DEGs and the HPS content. S1Table. The results of sequencing data quality for HP-WD and HP-TC. S2 Table. Summary of HP unigenes annotated in seven public databases. S3 Table. Identified the genes associated with polysaccharide biosynthesis, along with their Fragments Per Kilobase of transcript per Million mapped reads (FPKM) values. S4 Table. Pearson correlation analysis between DEGs and the HPS content.
(ZIP)
