# Supplementary material for: Effect of different origins on genes encoding key enzymes involved in the polysaccharide biosynthetic pathway in Hedysarum polybotrys Hand.-Mazz
Source: PLoS One. 2025 Apr 22;20(4):e0317890. doi: 10.1371/journal.pone.0317890 (PMC12013888; doi:10.1371/journal.pone.0317890)
Supplement: S1 File — S1 Fig. Standard curve of glucose at 490 nm. S2 Fig. Length Distribution of unigenes and transcripts. S3 Fig. Venn diagram of annotated unigenes from the different databases. S4 Fig. Gene Function Classification (GO) of HP. S5 Fig. KEGG pathway enrichment of the differential genes in HP-WD and HP-TC. S6 Fig. Pearson correlation analysis between DEGs and the HPS content. S1Table. The results of sequencing data quality for HP-WD and HP-TC. S2 Table. Summary of HP unigenes annotated in seven public databases. S3 Table. Identified the genes associated with polysaccharide biosynthesis, along with their Fragments Per Kilobase of transcript per Million mapped reads (FPKM) values. S4 Table. Pearson correlation analysis between DEGs and the HPS content. (ZIP) [file pone.0317890.s001.zip › Supporting Information - CompressedZIP File Archive/S4 Table.doc]

S4 Table. Pearson correlation analysis between DEGs and the HPS content. <https://doi.org/10.6084/m9.figshare.28180748.v1>

| **Gene ID** | ***r*** | ***P* value** |
| --- | --- | --- |
| GAE 2 | -0.9696 | 0.0014 |
| GMPP 1 | -0.9907 | 0.0001 |
| GMPP 2 | -0.8466 | 0.0335 |
| GPI 1 | 0.9323 | 0.0067 |
| GPI 2 | -0.9872 | 0.0002 |
| GPI 3 | -0.9570 | 0.0027 |
| HK 1 | -0.9799 | 0.0006 |
| HK 2 | -0.9896 | 0.0002 |
| HK 3 | -0.9759 | 0.0009 |
| HK 4 | -0.9753 | 0.0009 |
| HK 5 | 0.9820 | 0.0005 |
| HK 6 | 0.9792 | 0.0006 |
| INV 1 | -0.9279 | 0.0076 |
| INV 2 | -0.8849 | 0.0191 |
| INV 4 | 0.8925 | 0.0167 |
| MPI 1 | 0.9704 | 0.0013 |
| MPI 2 | -0.9780 | 0.0007 |
| MPI 3 | -0.8979 | 0.0151 |
| PGM 1 | -0.9804 | 0.0006 |
| PGM 2 | -0.9349 | 0.0062 |
| PGM 3 | 0.9880 | 0.0002 |
| PGM 4 | 0.9969 | 0.0000 |
| PMM 1 | 0.9765 | 0.0008 |
| PMM 2 | -0.9783 | 0.0007 |
| RHM 2 | -0.9755 | 0.0009 |
| SPS 1 | 0.9480 | 0.0040 |
| SPS 2 | 0.9805 | 0.0006 |
| SUS 1 | 0.9961 | 0.0000 |
| SUS 2 | -0.9708 | 0.0013 |
| SUS 3 | -0.9453 | 0.0044 |
| UGDH 2 | 0.9183 | 0.0097 |
| UGDH 3 | 0.9703 | 0.0013 |
| UGDH 4 | 0.9528 | 0.0033 |
| UGE 1 | -0.9329 | 0.0066 |
| UGE 2 | -0.9018 | 0.0140 |
| UGE 3 | -0.9641 | 0.0019 |
| UGE 4 | -0.9778 | 0.0007 |
| UGP2 1 | 0.9945 | 0.0000 |
| UGP2 2 | -0.8878 | 0.0182 |
| UGP2 3 | -0.9244 | 0.0084 |
| UGP2 4 | -0.9773 | 0.0008 |
| UGPase 1 | 0.9081 | 0.0123 |
| UGPase 2 | -0.9649 | 0.0018 |
| UGPase 3 | -0.9772 | 0.0008 |
| UGPase 4 | -0.9977 | 0.0000 |
